# Response to Cadmium in *Silene vulgaris* Ecotypes Is Distinctly Affected by Priming-Induced Changes in Oxidation Status of Macromolecules

**DOI:** 10.3390/ijms242216075

**Published:** 2023-11-08

**Authors:** Alina Wiszniewska, Mateusz Labudda, Ewa Muszyńska

**Affiliations:** 1 Department of Botany, Physiology and Plant Protection, University of Agriculture in Kraków, 31-120 Cracow, Poland; a.wiszniewska@urk.edu.pl; 2 Department of Biochemistry and Microbiology, Institute of Biology, Warsaw University of Life Sciences-SGGW, 02-776 Warsaw, Poland; mateusz_labudda@sggw.edu.pl; 3 Department of Botany, Institute of Biology, Warsaw University of Life Sciences-SGGW, 02-776 Warsaw, Poland

**Keywords:** bladder campion, DNA oxidation, lipid peroxidation, primed state, protein carbonylation, metallophyte

## Abstract

This study investigated the impact of several priming agents on metal-tolerant and sensitive *Silene vulgaris* ecotypes exposed to environmentally relevant cadmium dose. We analyzed how priming-induced changes in the level of lipid, protein, and DNA oxidation contribute to calamine (Cal) and non-calamine (N-Cal) ecotype response to Cd toxicity, and whether the oxidative modifications interrelate with Cd tolerance. In non-primed ecotypes, the levels of DNA and protein oxidation were similar whereas Cal Cd tolerance was manifested in reduced lipid peroxidation. In both ecotypes protective action of salicylic acid (SA) and nitric oxide (NO) priming was observed. SA stimulated growth and reduced lipid and DNA oxidation at most, while NO protected DNA from fragmentation. Priming with hydrogen peroxide reduced biomass and induced DNA oxidation. In N-Cal, priming diminished Cd accumulation and oxidative activity, whereas in Cal, it merely affected Cd uptake and induced protein carbonylation. The study showed that priming did not stimulate extra stress resistance in the tolerant ecotype but induced metabolic remodeling. In turn, the lack of adaptive tolerance made the sensitive ecotype more responsive to the benefits of the primed state. These findings could facilitate priming exploitation with a view of enhancing metallophyte and non-metallophyte suitability for phytoremediation and land revegetation.

## 1. Introduction

The toxicity and bioaccumulation of trace metals are emerging global issues adversely affecting all life forms. Among metals, cadmium (Cd) is a particularly dangerous element that can cause severe environmental and health risks. It has been ranked seventh by the Agency for Toxic Substances and Disease Registry, which assesses environmental health hazards related to exposure to both natural and human-made toxic substances [1]. For humans, Cd is a carcinogen (group 1) and can be incorporated into an organism by contaminated plant-based food [2]. Considering Cd persistence and omnipresence in the environment, its multifaceted toxicity for different trophic levels requires proper recognition. Particular emphasis is placed on Cd’s effect on plants since they are capable of absorbing and accumulating metals in the tissues, leading to a concentration of toxic elements, and their introduction to the food chains.

Cadmium phytotoxicity toward cell structures is related to the induction of oxidative stress. In this condition, reactive oxygen species (ROS) are overproduced, and antioxidant machinery is not efficient enough to neutralize their excess. Cd as a non-redox-active element—it does not form ROS directly but increases the amount of redox-active elements, most probably due to their replacement from enzyme-active sites. Cd also enhances ROS production by activating stress-specific oxidases in the cytoplasm and mitochondria (the latter being usually quiescent in this respect in unstressed tissues) [3]. ROS, together with reactive nitrogen species (RNS) including nitric oxide (NO), is the main cause of oxidative alterations in the structure of cellular components [4]. Under normal, nonstressful conditions, the majority of ROS are scavenged by the antioxidant system, while a small portion is utilized for signaling and modifications of macromolecules [5]. These proportions are disturbed under stress, and higher amounts of ROS interact with cellular macromolecules: proteins, nucleic acids, and membrane lipids. Oxidative modifications are usually associated with damage and loss of the function of the compound. Lipid and protein oxidation is often irreversible and indicates oxidative impairments/stress encountered by the cell and the whole organism [6]. Modifications to DNA are a priority subject to repair in order to avoid mutations [7], whereas oxidation of mRNA may constitute a post-translational regulation of gene expression [8]. Sometimes, as in the case of cell wall polysaccharides, oxidative modifications are not destructive and occur during normal plant development and organ growth, contributing to cell wall extension [9]. The products of lipid, protein, and nucleic acid oxidation are involved in cellular signaling and gene regulation, thus also playing a role in numerous developmental processes and stress responses [8,10].

Plant survival under stress caused by Cd toxicity may be ameliorated by priming. This term refers to the preconditioning with exogenously applied priming agent(s) (of physical, chemical, or biological character) in order to induce a considerable level of ‘stress awareness’ in the organism. In such primed state, plants acquire abilities to counteract the negative effects of stress and to sustain growth in unfavorable environments [11,12]. The most potent priming agents mitigating Cd stress effects are small signaling molecules: hydrogen peroxide (H_2_O_2_) and gasotransmitters such as nitric oxide (NO) and hydrogen sulfide (H_2_S), as well as salicylic acid (SA) [11]. Precise mechanisms of their priming action have been extensively investigated, and most of the research concludes that they are attributed to the intensification of antioxidant defense and ROS scavenging [13,14,15,16]. The priming agents also inhibit Cd accumulation, thus further reducing oxidative impairments [17,18]. On the other hand, little is known about the interconnection between priming and oxidation status of macromolecules in relation to the adaptive trait of trace metal tolerance. Hyperaccumulating and metal-tolerant species or ecotypes are seldom subjected to priming, so there is limited information on the specific features of their oxidative response to this kind of pretreatment. A study on Cd hyperaccumulator *Silene sendtneri* revealed that antioxidant activity increased only in some of the applied priming combinations, and, thus, it was not a versatile response [19]. Moreover, priming with synthetic auxin 2,4-dichlorophenoxyacetic acid (2,4-D) seemed to stimulate oxidase activity and peroxidation reactions in hyperaccumulating fern *Salvinia natans* [20]. Studies identifying specific reactions to priming in related tolerant and susceptible genotypes are also lacking. Such knowledge would facilitate our understanding of the role of both oxidation status and priming effects for innate tolerance to toxic metals. This study was conducted with the intention to fill, at least partially, this gap. Our aim was to compare the effect of Cd and previously applied priming on the oxidation level of critical cell macromolecules—lipids, proteins, and DNA—in two ecotypes of bladder campion (*Silene vulgaris* (Moench) Garcke). Ecotypes were selected for their distinct Cd tolerance: Cd-tolerant calamine ecotype and referential (sensitive), non-calamine one. We have also examined how priming contributes to Cd accumulation, ionic balance maintenance, and sulfur content. We hypothesized that the responses to applied Cd stress and pretreatment with various priming agents differ between the ecotypes and reflect their distinct strategies to cope with Cd toxicity. Distinguishing the responses common to both ecotypes and those that are ecotype-specific may contribute to understanding the mechanisms responsible for priming-induced tolerance to metallic stress.

## 2. Results

### 2.1. Growth Parameters

The micropropagation coefficient (MC), depicting proliferation efficiency and formation of new shoots, ranged from 7.5 to 11.4 in the N-Cal ecotype (Table 1). The MC of shoots primed with SA increased in comparison with non-primed shoots by 40%, while other priming agents had no significant impact on shoot proliferation ability in this ecotype.

The dry biomass of N-Cal shoots increased as a result of priming with NO (by 19%) and SA (by 27%) but decreased by 42% after H_2_O_2_ priming (Table 1). These three priming agents induced changes in shoot morphology. SA and NO-primed shoots were enlarged, particularly on the surface of the initial explant. Shoots primed with NO branched intensively, whereas, in shoots primed with SA and H_2_O_2_, changes in leaf blade morphology occurred (Table 1). H_2_O_2_ caused shoot chlorosis (Supplementary Materials Figure S1). Priming with ASA had no significant effect on shoot biomass accretion and morphology.

In the Cal ecotype, the MC of non-primed shoots amounted to 11.9 (Table 1). The MC increased after ASA and SA priming by 31 and 27%, respectively (Table 1). H_2_O_2_ and NO had no effect on this parameter. Shoot dry biomass was affected only in H_2_O_2_-priming cultures, where it decreased by 18%. Red coloration appeared on the leaves from this treatment. In shoots primed with SA internodes were elongated, while shoots primed with NO formed flowers. ASA priming did not cause any visible differences in shoot morphology in comparison with non-primed shoots.

A comparison of growth parameters between both ecotypes revealed that shoots of the Cal ecotype had higher proliferation ability and DW accretion than N-Cal shoots (Table 1). Only N-Cal shoots primed with NO multiplicated as efficiently as the majority of Cal shoots. Shoot proliferation in both ecotypes was stimulated by SA, while in the case of Cal, it was also stimulated by ASA. In both ecotypes priming with H_2_O_2_, SA, and NO caused alterations in shoot morphology, and H_2_O_2_ priming significantly reduced shoot biomass.

### 2.2. Enzymatic and Nonenzymatic Oxidation of Lipids

**Figure 1:**
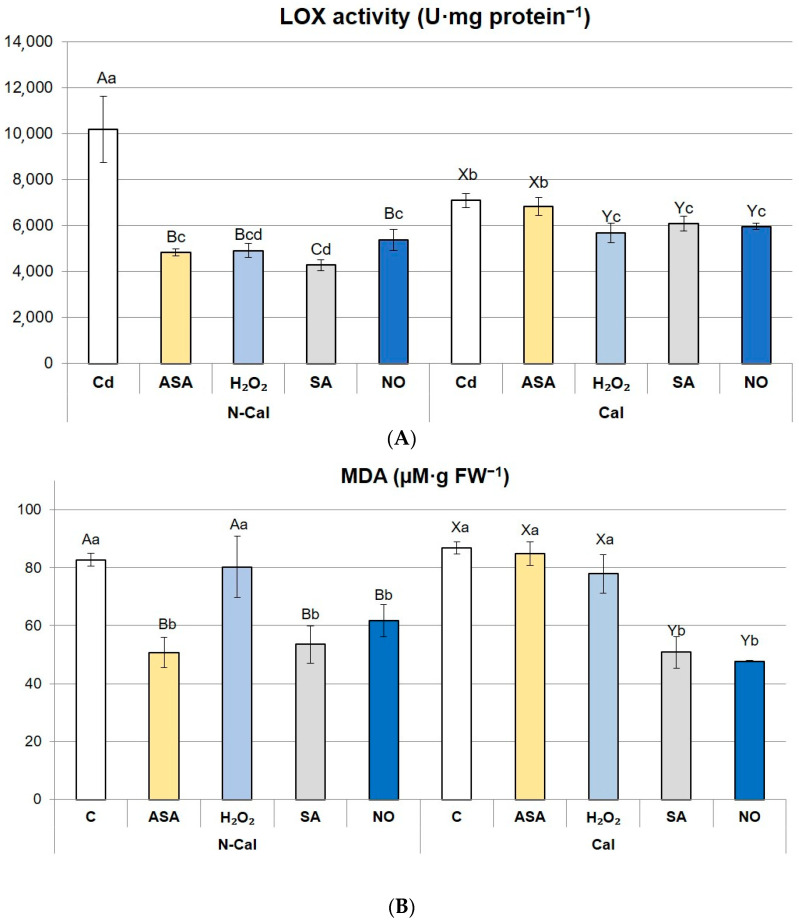
Activity of lipoxygenase (**A**) and malondialdehyde (MDA) content (**B**) in the primed shoots of non-calamine (N-Cal) and calamine (Cal) ecotypes of *Silene vulgaris.* Significant differences between treatments within each ecotype are marked with capital letters (A–C for N-Cal, X–Y for Cal), whereas significant differences between treatments are marked with lowercase letters.

Enzymatic oxidation of lipids was evaluated on the basis of LOX activity. In non-primed shoots exposed to Cd^2+^, LOX activity was 1.5-fold higher in N-Cal than in the Cal ecotype (*p* < 0.05) (Figure 1A). In N-Cal, LOX activity declined after the application of each priming agent. The highest reduction, amounting to 58%, occurred in SA-primed shoots (Figure 1A). In other priming treatments: ASA, H_2_O_2,_ and NO, LOX activity dropped nearly by half (48–53% (*p* > 0.05)). In the Cal ecotype, LOX activity dropped by 14–20% in the shoots primed with H_2_O_2_, SA, and NO, in comparison with non-primed cultures (Figure 1A). Priming with ASA did not affect LOX activity in this ecotype.

The content of malondialdehyde (MDA), a product of nonenzymatic lipid peroxidation, was the same in non-primed N-Cal and Cal shoots (Figure 1B). In both ecotypes, priming with H_2_O_2_ did not affect MDA concentration, whereas SA and NO reduced it (drop by 25–35% and 42–45% in N-Cal and Cal, respectively, *p* > 0.05) (Figure 1B). Priming with ASA caused a significant MDA decline in N-Cal, while in the Cal ecotype, it had no effect on this parameter (Figure 1B).

### 2.3. Assessment of DNA Damage and Excision of Oxidized Bases by Repair Enzyme

**Figure 2:**
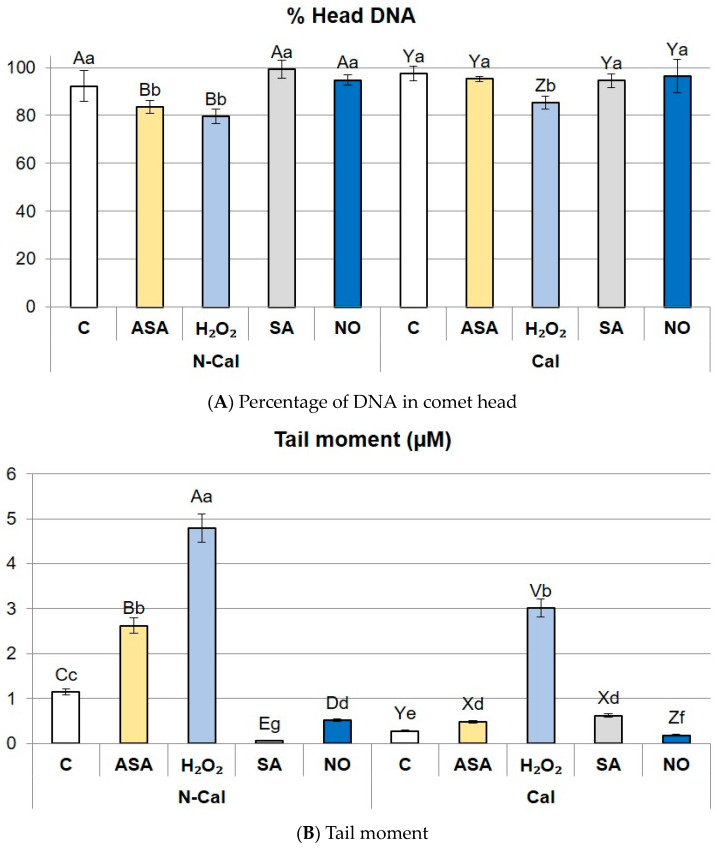

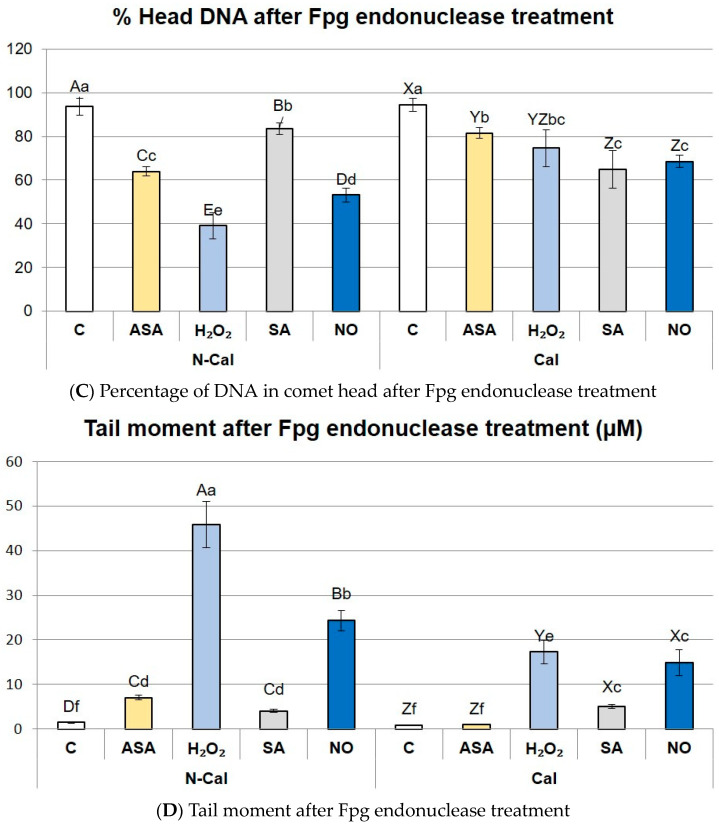
Total DNA damage (**A**,**B**) and oxidative DNA damage (**C**,**D**) in the primed shoots of non-calamine (N-Cal) and calamine (Cal) ecotypes of *Silene vulgaris.* Significant differences between treatments within each ecotype are marked with capital letters (A–E for N-Cal, V–Z for Cal), whereas significant differences between treatments are marked with lowercase letters.

Assessment of DNA integrity by the comet assay revealed that applied Cd^2+^ concentration did not induce serious DNA fragmentation in *S. vulgaris* ecotypes (Figure 2A,B). Without priming, in both ecotypes, the percentage of DNA in the comet head (% head DNA), depicting undamaged genetic material, exceeded 90% (Figure 2A), and tail moment (TM) was low (1.1 and 1.5 µm in N-Cal and Cal ecotype, respectively (*p* > 0.05) (Figure 2B). Priming with H_2_O_2_ significantly diminished DNA content in the comet head and increased the TM parameter, irrespective of the ecotype (Figure 2A,B). Differential response of the ecotypes concerned priming with ASA, which enhanced DNA fragmentation only in the N-Cal ecotype (Figure 2A,B). Priming with SA and NO did not induce DNA damage in comparison with non-primed shoots. Observed increments in DNA damage were not pronounced, and % head DNA did not drop below 80%.

Nuclei incubation with endonuclease Fpg (Fpg assay in short) was conducted to reveal the existence of oxidative modifications in DNA. An increase in DNA breakage, in relation to control comet assay performed without Fpg treatment, informed on the excision of oxidized bases from DNA strands.

Cd^2+^ treatment itself did not contribute to enhanced DNA oxidation as the level of DNA damage was comparable in non-primed shoots of both ecotypes, in relation to the results obtained without Fpg incubation (Figure 2C,D). In turn, priming treatments caused a decline in % head DNA in the Fpg assay, irrespective of the genotype (Figure 2C). Considering TM, only in ASA-primed Cal shoots, this parameter was the same as in non-primed shoots, whereas in the remaining treatments, it increased (Figure 2D). In the N-Cal ecotype, the highest level of base oxidation was detected after priming with H_2_O_2_ (39% head DNA, TM = 45.8 µm), followed by NO (53% head DNA, TM = 24.3 µm). Moderate levels of DNA oxidation occurred after ASA (64% head DNA, TM = 7.0 µm) and SA (83% head DNA, TM = 4.0 µm) treatments (Figure 2C,D). In the Cal ecotype DNA oxidation was less pronounced than in N-Cal; however, H_2_O_2_ and NO induced it the most (% head DNA 64.8 and 68.5%, respectively, and TM 17.3 and 14.8 µm, respectively).

### 2.4. Protein Content and Its Oxidation Status

**Figure 3:**
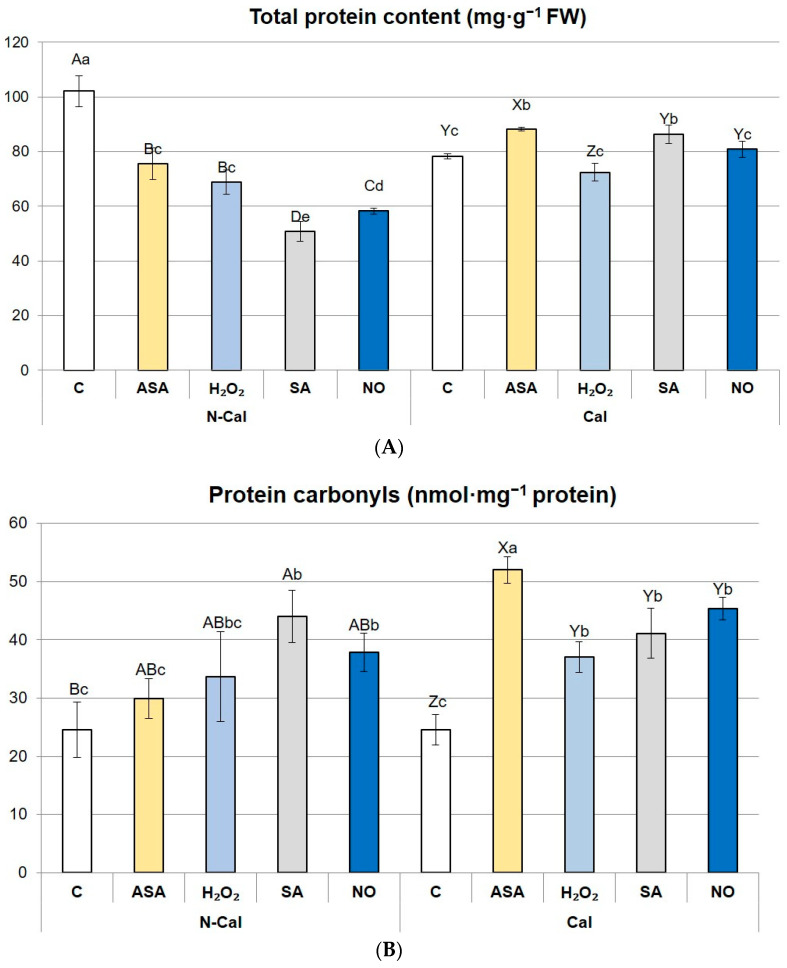
The content of total proteins (**A**) and their carbonylated derivatives (**B**) in the primed shoots of non-calamine (N-Cal) and calamine (Cal) ecotypes of *Silene vulgaris.* Significant differences between treatments within each ecotype are marked with capital letters (A–D for N-Cal, X–Z for Cal), whereas significant differences between treatments are marked with lowercase letters.

Total protein content in non-primed shoots grown in the presence of Cd^2+^ was higher in N-Cal than in the Cal ecotype (Figure 3A). Priming differentially affected the accumulation of soluble proteins in the ecotypes. In N-Cal, protein content declined in each priming treatment. The weakest declines were caused by ASA and H_2_O_2_ (by 26–22% in comparison with non-primed shoots, *p* > 0.05 among these two treatments), while the highest occurred after priming with SA (by 51% in comparison with non-primed control) (Figure 3A). In the Cal ecotype, priming with ASA and SA caused an increase in protein content whereas the application of H_2_O_2_ and NO did not affect the level of proteins significantly (Figure 3A).

The concentration of total protein-bound carbonyls (sum of aldehyde and ketone derivatives) was similar in non-primed shoots of both ecotypes (Figure 3B). In the N-Cal ecotype, the carbonylation significantly increased as a result of SA and NO priming (Figure 3B). Other treatments did not affect this parameter in comparison with non-primed N-Cal control (Figure 3B). In the Cal ecotype, carbonylation was enhanced irrespective of the applied priming agent. The highest increase in the content of carbonyl derivatives was an effect of ASA priming.

Regarding a profile of carbonyl derivatives, aldehydes prevailed over ketones in both ecotypes, constituting 63.8–69.0% of total carbonyls (Table 2). In the N-Cal ecotype, a proportion of aldehydes and ketones was not affected by priming treatments, whereas in the Cal ecotype, all priming agents induced ketone formation in comparison with non-primed control (Table 2).

### 2.5. Cadmium Accumulation

**Figure 4:**
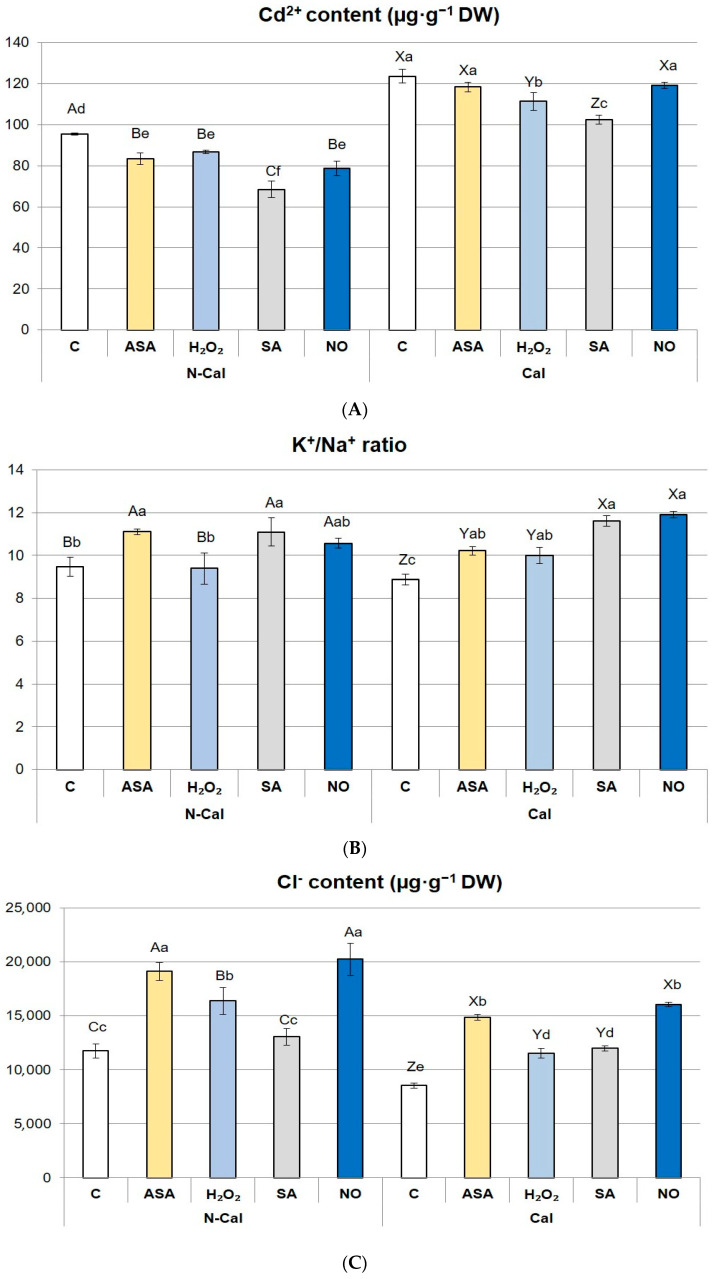

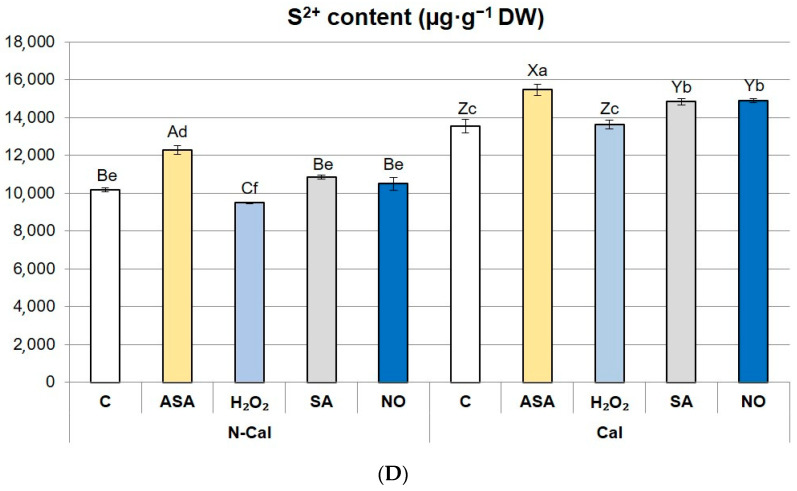
The ion content in the primed shoots of non-calamine (N-Cal) and calamine (Cal) ecotypes of *Silene vulgaris.* (**A**) Cd^2+^, (**B**) K^+^/Na^+^ ratio, (**C**) Cl^−^, (**D**) S^2+^. Significant differences between treatments within each ecotype are marked with capital letters (A–C for N-Cal, X–Z for Cal), whereas significant differences between treatments are marked with lowercase letters.

All priming agents reduced Cd^2+^ accumulation in the N-Cal shoots in comparison with non-primed control (Figure 4A). The most pronounced decrease (by 29%) occurred after SA priming. In the Cal ecotype, H_2_O_2_ and SA priming inhibited Cd^2+^ accumulation while other treatments did not affect it. Similar to N-Cal, in the Cal ecotype SA priming reduced Cd^2+^ accumulation the most (by 18%). Comparing both ecotypes, Cal has a significantly higher ability to accumulate Cd than N-Cal. Non-primed Cal shoots contained nearly 30% more Cd than respective N-Cal shoots.

### 2.6. Ionic Balance

K^+^/Na^+^ ratio, an indicator of ionic balance, increased by 17% in the N-Cal ecotype after priming with ASA and SA, in comparison with non-primed control. It also increased as a result of priming with all tested agents in the Cal ecotype, by 12–34%, depending on the treatment (Figure 4B).

Chloride content in non-primed shoots was higher by 37% in N-Cal than in Cal. In N-Cal shoots Cl^−^ accumulation increased after priming with ASA (by 62%), NO (by 72%), and H_2_O_2_. In the Cal ecotype, all priming agents significantly increased Cl^−^ content. In the shoots primed with respective compounds, Cl^−^ content was always higher in N-Cal than in the Cal ecotype (Figure 4C).

The sulfur content was 33% higher in non-primed shoots of Cal than the N-Cal ecotype. In the case of N-Cal, ASA priming elevated S^2+^ content, H_2_O_2_ declined it, while SA and NO did not affect it. In turn, priming with ASA, SA, and NO further enhanced S^2+^ accumulation in Cal shoots (Figure 4D).

### 2.7. Principal Component Analysis (PCA)

**Figure 5:**
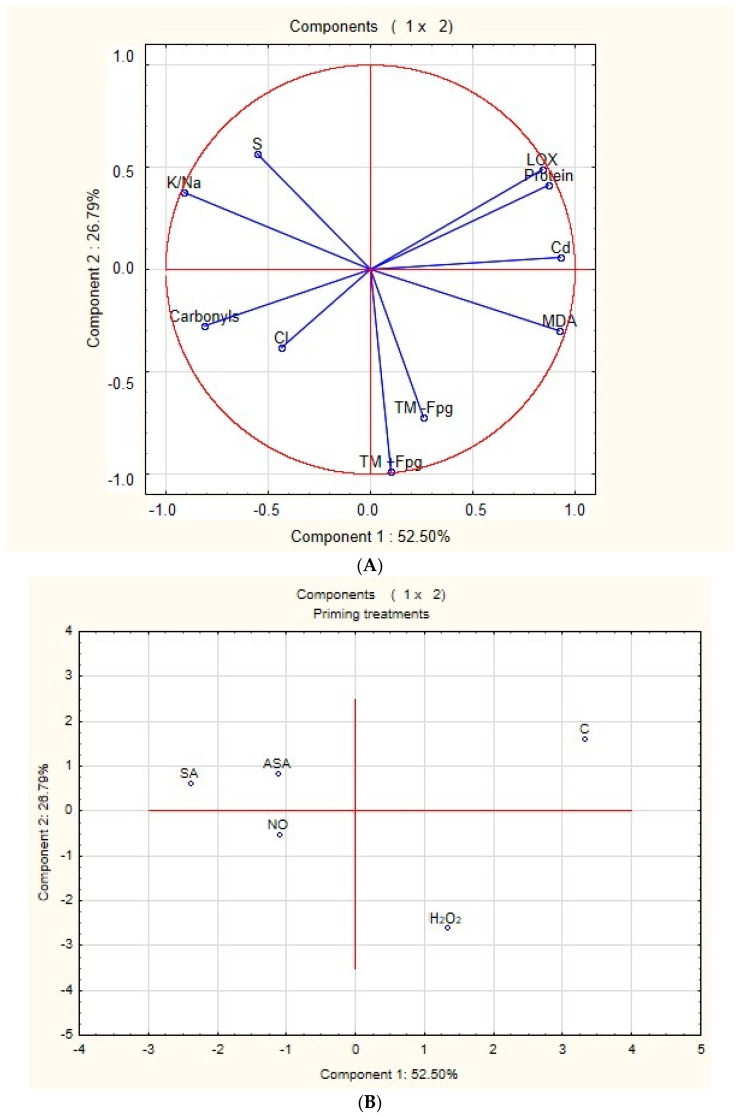

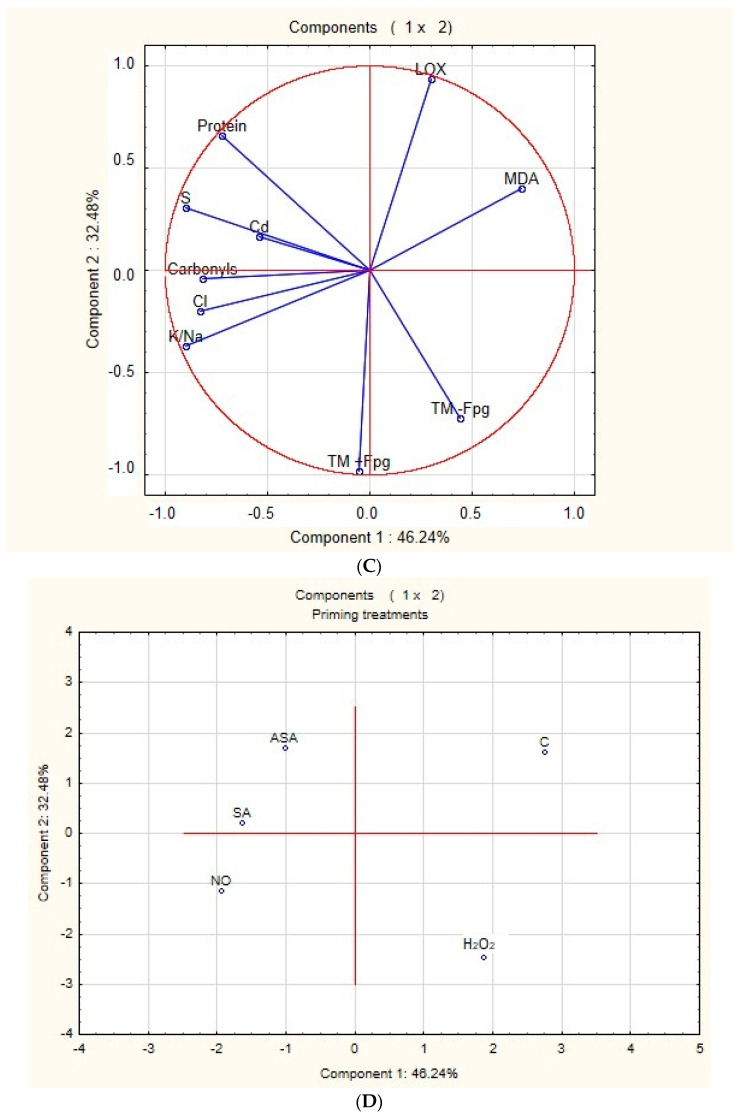
The results of principal component analysis conducted for two *S. vulgaris* ecotypes of contrasting metal tolerance (N-Cal—non-calamine, sensitive; Cal—calamine, tolerant) subjected to Cd exposure and priming. ASA—acetylsalicylic acid, H_2_O_2_—hydrogen peroxide, SA—salicylic acid, NO—nitric oxide (NO). C stands for control treatment (hydropriming). (**A**) Variables and (**B**) treatments correlated with the main components of the total variance in the N-Cal ecotype. (**C**) Variables and (**D**) treatments correlated with the main components of total variance in the Cal ecotype.

Principal component analysis was conducted to determine and group variables responsible for observed responses to respective priming treatments in both *S. vulgaris* ecotypes. In the N-Cal ecotype, PCA distinguished two main components that explained 79.29% of the total variance (TV). The first component (52.50% TV) was associated with differential oxidation status of lipids and proteins and ionic balance affected by accumulation of Cd^2+^ ions. Positively correlated with this component were: Cd^2+^ content in the shoots, LOX activity with MDA content (enzymatic and nonenzymatic lipid peroxidation), and total protein content (Figure 5A). Variables negatively correlated with component 1 included protein carbonyl content (protein oxidation) and K^+^/Na^+^ ratio (ionic balance indicator). Component 2 (26.79% TV) was related to the integrity of genetic material since strong negative correlations with this component were determined for both oxidative and total DNA damage, expressed by the TM parameter (Figure 5A). Considering priming treatments and distinguished principal components, component 1 correlated positively with the treatment causing excessive lipid peroxidation (non-primed control) and negatively with the treatments inducing oxidative changes in proteins (priming with ASA, SA, and NO). Component 2 correlated negatively with H_2_O_2_ priming treatment as it was the variant that enhanced DNA fragmentation and oxidation (Figure 5B).

In the Cal ecotype, the first two principal components explained 78.72% of the TV. Component 1 (46.24%) was associated with differential oxidation of lipids and proteins, ionic balance, and nutrition status of cultured shoots. Unlike in N-Cal, cadmium accumulation was not correlated with any of the main components. Component 1 positively correlated with MDA content (nonenzymatic lipid peroxidation). Negative correlations occurred between component 1 and protein carbonyls, total protein content, K^+^/Na^+^ ratio, and Cl^−^ and S^2+^ contents. The second component (32.48% TV) reflected the impact of priming on lipid and DNA damage. The activity of the LOX enzyme was positively correlated with this component and negatively correlated with both parameters depicting DNA integrity (TM + Fpg and TM-Fpg) (Figure 5C). Considering correlations between the experimental treatments and distinguished principal components, the pattern was similar to the one distinguished for the N-Cal ecotype. With component 1, control treatment (‘non-priming’) was positively correlated, while the treatments that improved ionic balance and nutrition status (ASA, SA, and NO)—negatively. Component 2 was positively correlated with ASA and C treatments (treatments that did not promote DNA fragmentation) and negatively correlated with H_2_O_2_ treatment (treatment that enhanced DNA oxidation and fragmentation) (Figure 5D).

## 3. Discussion

Contamination of the environment with metallic elements is among the most significant challenges to plants’ metabolic activity leading either to its disturbance or adjustment. Metal-bearing sites, as very restrictive habitats for plants, stimulate strong modifications in cellular processes, resulting in the natural selection of plant communities adapted to metals, called metallophytes. These may be the genetically altered ecotypes of species found in uncontaminated areas or taxa capable of surviving only in the presence of metals [21]. Regardless of the classification, metallophytes exhibit unique functional traits for handling excess amounts of metallic ions. In this study, we examined reactions to long-term Cd exposure preceded by short-term priming in two bladder campion (*S. vulgaris*) ecotypes: metal-adapted and non-adapted. The species is a pseudometallophyte that easily forms ecotypes tolerant to a wide range of metallic elements, also common in nonpolluted areas [22]. The adapted ecotype used in this study was a calamine one, naturally inhabiting the metal-polluted area of the Olkusz Ore Bearing Region (Poland). The soils in this area contain elevated concentrations of Zn, Cd, Pb, and As ions [23]; therefore, calamine ecotype exhibits a wide spectrum of tolerance to metal elements. The ecotype activates ubiquitous reactions to counteract their toxicity, including efficient ROS scavenging, and, also, produces a considerable amount of ROS for signaling purposes [22,24]. The second ecotype used in this study, the non-metallicolous one, was found to be sensitive to Cd, Ni, Pb, and Zn [22,24]. In this study, we tested how the contrasting ecotypes respond to Cd after priming with various priming agents and how priming affects the oxidative status of macromolecules, Cd accumulation, and ionic balance. Following induction of the primed state, shoots were exposed to Cd at a concentration that corresponds to the level determined for the soil in the calamine area (16 µM CdCl_2_).

### 3.1. Ecotypes’ Growth Characteristics Were Similarly Modulated via Priming

In both ecotypes, the formation of new shoots was promoted by SA treatment, which may be related to the reduced Cd accumulation as a result of SA priming. It can be explained by a decrease in the pool of available transport proteins resulting from an interrelation between exogenous SA and an expression of uptake/transport-related genes [25]. However, other studies showed that SA impact on Cd accumulation is dose dependent. In *Silene sendtneri*, priming with a higher SA dose (1.0 mM) boosted Cd uptake and translocation to the extent noted only for hyperaccumulating plants [19]. Massive Cd accumulation deteriorated growth, but primed plants survived under such Cd concentration that was lethal to non-primed plants. Interestingly, an improvement of growth parameters in the Cal ecotype was also achieved after ASA treatment although Cd content in the shoots did not decline. It suggests the existence of a specific defense mechanism in the calamine ecotype that allows for sustaining undisturbed organogenesis at high Cd concentration in the tissues. The acetylated derivative of salicylic acid is often regarded as a functional analog of SA [26], but our study indicates that the distinct biological activity of ASA should also be considered.

Uniform ecotypes’ reactions also included a significant reduction of dry biomass after H_2_O_2_ priming. This finding is in contrast to other studies where exogenous H_2_O_2_ induced dry biomass accretion in plants exposed to Cu and Cr [27,28], most probably due to the protection of photosynthetic apparatus. As our experiment was held in in vitro conditions, biomass accretion in *S. vulgaris* ecotypes was unlikely to be based on photosynthetic activity [29]. Instead, lower dry matter content could be associated with higher hydration status of cultured shoots. Oxidative burst generated by H_2_O_2_ treatment may lead to hyperhydricity, related to the accumulation of ROS in the apoplast, and disturbances of cell ultrastructure [30], affecting the overall growth performance of plantlets.

### 3.2. Oxidation Status in Non-Primed Shoots Treated with Cd

The level of lipid peroxidation was evaluated on the basis of LOX activity (enzymatic oxidation) and MDA accumulation (nonenzymatic oxidation). This is a simplified classification because some part of MDA is also produced in an enzymatic reaction [31]. Although both processes occur side by side and affect each other [32], we have detected differential responses between the ecotypes in relation to enzymatic and nonenzymatic oxidation. The activity of LOX was much higher in non-primed N-Cal than in Cal shoots, while MDA content was the same. It suggests that the enzymatic pathway of lipid oxidation was suppressed in the Cd-tolerant ecotype and induced in the non-tolerant one. In contrasting *Sedum alfredii* ecotypes, a similar pattern occurred under higher doses of Pb ions: MDA content was comparable while the LOX activity increased only in the non-accumulating ecotype [33]. Such differences in the LOX activity may reflect the altered composition of cellular membranes in relation to the content and profile of polyunsaturated fatty acids (PUFA) in ecotypes tolerant and susceptible to abiotic stresses [34]. A particular level of membrane fluidity and permeability, conditioned by the specific lipid composition, was found to be crucial for the development of Cd tolerance and hyperaccumulation in *Noccaea caerulescens* [35]. Differences in the LOX activity may be also associated with the synthesis of signaling compounds, mainly oxylipins, from lipid peroxides generated by LOX [36,37]. It is noteworthy that MDA content did not differ in *S. vulgaris* ecotypes. This compound, commonly regarded as a marker of oxidative stress [6], does not seem to be such an undisputable indicator in *S. vulgaris*. Our previous studies revealed that various ecotypes and populations of this species are often capable of maintaining stability of or reducing the MDA content regardless of stress exposure [38,39]. Similarly, simultaneous treatment of the serpentine ecotype with Zn, Pb, and Cd in the concentrations reflecting their contents in post-industrial substrate did not alter MDA content in comparison with untreated control and the calamine ecotype [24]. As there is evidence that MDA itself is a nontoxic product but contributes to further oxidative damage in other macromolecules [31], its ambiguous role in stress response in metal-tolerant ecotypes of *S. vulgaris* requires further investigation.

We assessed cadmium-induced DNA damage in the ecotypes twice: in nuclei directly isolated from Cd-treated shoots and in nuclei treated with Fpg endonuclease, an enzyme that recognizes an oxidized form of guanine (8-oxoguanine) and cuts off modified base. The first assay was addressed to estimate total DNA integrity, while the second assessed the level of oxidative modifications to nucleotides. As Cd binds only weakly to DNA, vast genotoxic effects of this metal are mediated via overproduced ROS, and these effects should be manifested in the second assay. However, at low micromolar concentrations, Cd may interact directly with proteins of the DNA repair system [40], which should be reflected in total DNA integrity. In non-primed *S. vulgaris* shoots, total DNA damage was slightly higher in the N-Cal ecotype than in Cal. Higher resistance of the Cal ecotype to DNA damage could be attributed to an adaptive response, a phenomenon involving an adaptation to the presence of genotoxic factor due to constant exposure to low doses of this or other genotoxin [41]. The stability of genetic material in the presence of trace metals usually indicates higher plant tolerance to their toxicity, as revealed by comparison of *Zygophyllum fabago* populations from Pb-contaminated and Pb-free sites [42] and *Solanum nigrum* treated with Cu [43]. Such ecotype-specific DNA damage response in *S. vulgaris* was also evidenced previously at lower Cd doses [39]. Considering oxidative DNA damage, both ecotypes responded similarly. It can be concluded that although the ecotypes are equally sensitive to base oxidation by ROS, mechanisms of DNA repair are likely more efficient in the metal-tolerant ecotype. Additionally, distinct patterns of epigenetic modifications between tolerant and non-tolerant ecotypes could influence general DNA damage response, as in Ni hyperaccumulator *Noccaea caerulescens* and its relative species *Arabidopsis thaliana* [44]. Particularly methylation of cytosine is often affected by oxidative modifications in the DNA, causing changes in gene expression and altering epigenetic profile [5].

Differences between the ecotypes involved altered protein accumulation. Cd-exposed Cal accumulated about 20% less protein than N-Cal. Usually, the protein content drops in sensitive plants as a result of metal treatment, in comparison with their metal-tolerant counterparts. This contrasting result could be attributed to the stimulation of rapid stress defense in N-Cal by enhanced synthesis of specific protective proteins. It was revealed that low tolerance to metal stress was associated with massive synthesis of heat shock proteins (HSP) in the cell lines of *S. vulgaris* and *Lycopersicon peruvianum* [45]. Upregulation and enhanced accumulation of various defense proteins, such as chaperones, metal-binding proteins, and enzymes catalyzing the synthesis of chelating peptides, as well as proteins involved in detoxification, were reported in plants subjected to Cd stress [46]. In turn, metal-tolerant species and ecotypes are capable of maintaining stable protein concentration under a wide range of metal concentrations [47,48], suggesting that the activity of the defense system is constitutively boosted. Interestingly, total protein carbonylation and the content of protein-bound aldehyde and ketone groups were comparable between both *S. vulgaris* ecotypes. It may suggest that proteins of both ecotypes are similarly susceptible to post-translational modifications. It is in accordance with our previous study on *S. vulgaris*, which indicated that shoots of non-metalliferous, calamine, and serpentine ecotypes untreated with metals had similar content of carbonylated proteins, and the application of Zn, Pb, and Cd ions did not contribute to changes in this parameter in metalliferous shoots [49]. However, the determined amounts of protein-bound carbonyls should be considered high in relation to the optimal level equal to 4 nmol carbonyl groups (C=O) per mg of proteins [50]. We assume that pronounced protein carbonylation could be associated with shoot aging resulting from prolonged in vitro cultivation [51].

### 3.3. Effect of Priming on Macromolecule Oxidation under Cd Treatment

Both ecotypes similarly responded to priming in relation to lipid peroxidation and DNA oxidation, indicating that these modifications are constitutively recognized by the species metabolic machinery and are not directly related to acquired tolerance to Cd toxicity. However, the ecotypes differed in the intensity of the reaction, and the levels of lipid and DNA oxidation were less harmful in the Cal ecotype. In turn, the ecotypes responded distinctly to priming agents in relation to the oxidation of proteins, indicating that priming interrelates with the protein-based mechanisms of conditioning adaptation and Cd tolerance in the studied species.

The main role of priming is an induction of mild defense response prior to stress occurrence [52]. In the case of metal stress, such defense relies mainly on the activation of antioxidant machinery and osmotic adjustment and contributes to altered abilities to uptake toxic ions [11]. In the current study, such ameliorative effects of priming were achieved in the non-tolerant *S. vulgaris* ecotype. First, all priming treatments reduced Cd accumulation, which, according to PCA, was correlated with the main component of variance explaining the response of this ecotype to priming. Lower Cd content in the tissues contributed to higher survival and improved growth of shoots. Second, applied priming agents either diminished or stabilized an oxidative activity and the resulting level of lipid and protein oxidation. These observations indicate that ROS were successfully scavenged in the majority of primed N-Cal plants. The least efficient was H_2_O_2_, which was incapable of reducing the MDA content and caused pronounced DNA damage and oxidation. Hydrogen peroxide is a strong oxidative agent itself and affects DNA integrity directly [53]. In addition, a common type of DNA damage results from the conjugation of MDA with guanine [5]. It seems that in N-Cal, unbalanced oxidative activity of exogenous H_2_O_2_ prevailed over the mechanisms controlling ROS homeostasis. Our findings are in contrast to the reports presenting the ameliorative effect of H_2_O_2_ priming on antioxidant response. The reaction may be, therefore, influenced by the dose and treatment time as the delicate balance between H_2_O_2_ production and scavenging may be easily disturbed [54].

In the Cal ecotype, the effects of priming were more contradictory. Only H_2_O_2_ and SA restricted Cd uptake, which was in accordance with the mode of their protective action reported in other studies [17,55]. However, PCA analysis showed that Cd accumulation was not correlated with any of the main components involved in Cal response to priming. Oxidation of lipids and total DNA damage was reduced the most by priming with SA and NO. These priming agents were previously reported as capable of protecting cellular components by increasing antioxidant capacity in plants treated with metals. Their beneficial action was manifested by reduced lipid peroxidation in common bean (*Phaseolus vulgaris*) exposed to As [56] and flax (*Linum usitatissimum*) treated with Cd [13], as well as by the protection of DNA from fragmentation [57,58]. However, NO may also directly interfere with DNA structure, leading to its fragmentation or base modification [59,60]. Such a phenomenon was also observed in the case of the Cal ecotype, where the level of oxidative changes in DNA was significantly elevated after NO priming, although without influence on growth parameters. Here, DNA oxidation could be an indicator of epigenetic changes that could alter cellular metabolism [61]. Accumulation of oxidized guanine affects epigenetic stability, leading to genome hypomethylation and distinct affinity of DNA damage sensor proteins [62,63]. As it may further influence plant adaptability to environmental challenges [62], the occurrence of such mechanisms in the metallicolous ecotype is plausible.

The most substantial difference between the ecotypes’ response to priming was the level of protein carbonylation. It increased in Cal, regardless of the applied agent. Considering the lack of growth deterioration and the stable pool of total proteins, pronounced carbonylation could be regarded as post-translational modification related to proteome rearrangement, in which proteins dedicated to degradation would be marked by carbonyl groups [6,51]. Proteome reconstruction could be directed toward the degradation of existing proteins in order to supply amino acids for the production of a battery of defense compounds: antioxidant enzymes, metal-binding proteins, peptides, or proteins interacting with DNA. One of the main targets of modifications driven by reactive oxygen and nitrogen species is antioxidant enzymes [64]. The assumption concerning Cal proteome rearrangement due to priming is supported by an increase in the content of S and Cl. Sulfur is essential for thiol synthesis, and it was evidenced that these compounds, including glutathione and phytochelatins, determine metal tolerance in the *Silene* genus [22,39,65]. In the presence of high doses of accumulated Cd, metabolism in Cal could be redirected toward increased synthesis of Cd-binding peptides and proteins. Elevated content of Cl ions, known to affect nitrogen mobilization [66] and protein biosynthesis [67], may further support proteomic alterations. These, together with potential epigenetic changes, likely ensure the protection of lipids from excessive peroxidation, preventing cell membranes from destabilization. The undisturbed structure of membranes enables the maintenance of optimal osmotic conditions in the cells, as indicated by a high ratio of ionic balance indicator K^+^/Na^+^ in the Cal ecotype. A similar protective mechanism was attributed to melatonin pretreatment in Cd-exposed mallow (*Malva parviflora*) plants [68].

## 4. Materials and Methods

### 4.1. Plant Material, Experimental Design, and Assessment of Growth Parameters

Two *Silene vulgaris* (Moench) Garcke) ecotypes were propagated in in vitro culture. Stock cultures were established from the seeds collected from the calamine area of Olkusz Ore Bearing Region (Poland; 50°17′ N, 19°30′ E) (calamine ecotype—Cal) and from uncontaminated forest clearing located in Zielonka, close to Warsaw (Poland; 52°28′ N, 21°25′ E) (non-calamine ecotype—N-Cal).

Prior to the priming experiment, cultures were maintained on basal propagation medium consisting of MS salt and vitamins [69] and supplemented with 0.65 g/L calcium gluconate (Sigma-Aldrich, Saint Louis, MO, USA), 0.5 g/L polyvinylpyrrolidone (PVP) (Sigma-Aldrich), 0.8% agar (Merck, Darmstadt, Germany), 4.4 µM 6-benzylaminopurine (BAP) (Sigma-Aldrich, Saint Louis, MO, USA), and 1.14 µM indole-3-acetic acid (IAA) (Sigma-Aldrich, Saint Louis, MO, USA). Medium pH was adjusted to 5.7.

For test culture establishment, 10 mm long apical parts of shoots were explanted onto Cd-free basal medium and submerged with sterile 0.1 mM water solutions of priming agents: acetylsalicylic acid (ASA), hydrogen peroxide (H_2_O_2_), nitric oxide (using sodium nitroprusside (SNP) as NO donor)), and salicylic acid (SA). In each individual Magenta box (Magenta LLC, Lockport, IL, USA), 5 mL of priming agent solution was poured onto 50 mL of solidified medium prior to culture initiation. As a control treatment, hydropriming with distilled water was applied. The media and distilled water were sterilized by autoclaving whereas solutions of priming agents were filter sterilized.

After 7 days of priming, shoots were transferred onto basal medium containing 16 µM CdCl_2_ and kept for 6 weeks in a growth chamber at 22 °C under a 16 h photoperiod (irradiance 80 μmol m^−2^s^−1^).

Data on growth and biochemical responses were collected after 6 weeks. Growth performance was evaluated on the basis of the number of newly produced adventitious shoots per explant expressed as micropropagation coefficient (MC) and dry biomass accretion (DW). Specific changes in shoot morphology were monitored.

### 4.2. Biochemical Analyses

#### 4.2.1. Malondialdehyde (MDA) Content

Malondialdehyde level was measured after the homogenization of 100 mg of shoots with 80% methanol. Samples were centrifuged (4 °C, 16,000× *g*, 20 min) and particular methanolic extracts were mixed with 0.5% 2-thiobarbituric acid dissolved in 20% trichloroacetic acid solution. Then, samples were incubated at 90 °C for 20 min, and reactions were stopped in an ice bath. After the next centrifugation of samples (16,000× *g*, 10 min), the absorbance of supernatants was measured at 440 nm, 532 nm, and 600 nm on a Varioskan LUX Multimode Microplate Reader (Thermo Scientific, Waltham, MA, USA). MDA content was calculated according to Hodges et al. [70].

#### 4.2.2. Lipoxygenase (LOX) Activity

Plant samples (300 mg) were homogenized on ice in 5 mL cold phosphate buffer (pH = 6.8), and homogenate was centrifuged at 15,000 rpm for 20 min at 4 °C. Reaction substrate, linoleic acid, was emulsified in phosphate buffer (pH = 6.0) according to Salcedo et al. [71]. Lipoxygenase activity was determined by spectrophotometric measurement of conjugated dienes at 234 nm, according to Gardner [72]. The reaction mixture consisted of reaction buffer (buffer pH assuring the highest enzyme activity was optimized prior to the analysis), plant extract, and linoleic acid. The calculation of LOX activity was made according to the equations provided by Gardner [72]. Protein content in plant extracts was determined according to Bradford [73]. LOX activity was expressed on the basis of protein content.

#### 4.2.3. Protein Carbonylation

Protein carbonylation level was estimated according to Muszyńska and Labudda [49]. Briefly, 100 mg of shoots were homogenized in an ice-cold 50 mM 3-(N-morpholino) propane sulfonic acid buffer (pH 7.2) containing 5 mM 2-mercaptoethanol, 2% polyvinylpyrrolidone, 5 mM CaCl_2_, and 0.5% Triton X-100. Homogenates were centrifuged (16,000× *g*, 4 °C, 20 min) and extracts were collected. The total soluble protein content was measured using Bradford [73]. Then, 2,4-dinitrophenylhydrazine in 2.5 M HCl was mixed with each extract before the incubation for 1 h (with shaking every 10 min) at room temperature in darkness. Individual blank protein samples were incubated only in 2.5 M HCl. Next, ice-cold 20% (*w*/*v*) trichloroacetic acid was added and samples were incubated on an ice bath for 5 min and centrifuged (16,000× *g*, 4 °C, 10 min). Pellets were washed 4 times with cold ethanol/ethyl acetate mixture (1:1, *v*/*v*), then solubilized in 6 M guanidine hydrochloride solution, and centrifuged again (16,000× *g*, 4 °C, 10 min). After warming up to room temperature, the absorbance was measured at 370 and 430 nm for the determination of aldehyde and ketone derivatives, respectively. Their content was calculated using a molar absorption coefficient for aliphatic hydrazones of 22,000 M^−1^ cm^−1^ and expressed in nmol of particular carbonyl groups per mg of protein. The sum of the content of aldehyde and ketone derivatives means the total protein-bound carbonyl groups.

### 4.3. Determination of DNA Oxidative Damage

DNA oxidative damage was assessed using comet assay modification based on the protocol of Collins and Azqueta [53]. Nuclei were isolated in a cold Tris-HCl buffer from 3 leaves from 3 randomly selected microshoots in each treatment. Nuclei were suspended in low-melting-point (LMP) agarose (Roth, Karlsruhe, Germany), and the suspension was applied to three wells on the Fragment Length Analysis Using Repair Enzymes (FLARE^®^) slide glass (Trevigen, BioTechne, Minneapolis, NE, USA). After agarose solidification, specimens were soaked in FLARE^®^ buffer for 5 min; afterward, a solution of formamidopyrimidine-DNA glycosylase (Fpg) from *E. coli* (Trevigen, BioTechne, Minneapolis, NE, USA) was added to two test wells on the slide. One well on each slide contained only the reaction buffer without the enzyme (control well). Slides were incubated in a thermostat at 37 °C in a humidity chamber for 30 min. Afterward, slides were subjected to pre-electrophoresis for DNA unwinding in cold (4 °C) alkaline buffer (pH > 13) for 20 min. Alkaline electrophoresis was conducted for 20 min at 18 V and 300 mA, followed by neutralization in a cold Tris-HCl buffer. Prior to analysis under epifluorescence in an AxioImager Multifunctional Microscope (Carl Zeiss, Jena, Germany), nuclei were stained with 4′,6-diamidino-2-phenylindole (DAPI). Images were analyzed in CaspLab 1.2.3beta2 software [74] and DNA damage was evaluated on the basis of % DNA in the comet head and tail moment parameter (TM = comet tail length·% DNA in tail (µm)). The assay was conducted independently three times, with a minimum of 60 nuclei scored per treatment in each experiment replication.

### 4.4. Determination of Cd, Cl, K, Na, and S Contents

For determination of Cd^2+^, Cl^−^, K^+^, Na^+^, and S^2+^ content, dry plant samples were treated with a mixture of nitric acid and perchloric acid (3:1 *v*/*v*) and analyzed using Thermo Scientific iCAP TQ ICP-MS spectrometer.

### 4.5. Statistical Analyses

Three independent experiments in three replicates were performed. Five Magenta boxes with five explants per experimental treatment constituted a replicate. Data were verified for normality and statistically analyzed using STATISTICA 13.0 software (StatSoft, Tulsa, OK, USA): one-way ANOVA to assess differences between the responses to applied priming agents within respective ecotype and two-way ANOVA to assess differences between ecotypes and treatments. Post hoc comparisons were conducted using the Tukey test. PCA analysis was conducted using the respective module in STATISTICA 13.0 software.

## 5. Conclusions

Our study revealed that the elevated concentration of Cd ions did not severely affect growth parameters but induced physiological alterations in *Silene vulgaris*. Considering the growth and oxidation of macromolecules, pronounced changes concerned metal-sensitive ecotypes more than the metal-tolerant ones. We confirmed that primed state induced before Cd exposure could mitigate oxidative impairments coinciding with metal treatment. However, the mode of priming agents’ action in defense response against metallic stress was ecotype-specific. While almost all tested priming agents improved the fitness of N-Cal specimens, the reaction of Cal was more ambiguous and involved the proteome remodeling and effective ROS scavenging rather than reduced metal accumulation. The results suggest that the application of priming agents on specimens with a certain level of adaptive tolerance may modify their reaction in an unpredictable way and not necessarily stimulate extra resistance to stress factors. It would be of importance in studies and practices that exploit metallophytes for phytoremediation. In contrast, the lack of adaptive tolerance trait makes metal-sensitive specimens more responsive to priming stimuli, intensifying their defense reaction. Therefore, priming application could be a beneficial approach in the case of numerous crop plants, particularly ornamental, cultivated for land revegetation. Since there are sparse studies comparing priming effects in related genotypes of contrasting stress tolerance, our study shed light on the mechanisms of their distinct susceptibility to external stimulation. It has been shown for the first time that responses to priming significantly differ in the ecotypes of distinct metal tolerance. The lack of adaptive tolerance made metal-sensitive ecotypes more responsive to the benefits of primed state induced prior to Cd exposure. In the tolerant ecotype, priming did not boost extra stress tolerance. In order to develop efficient methods preventing global environmental distortions, future research should focus on deciphering precise mechanisms underlying the operation of a battery of priming agents in taxa with different levels of tolerance to most challenging abiotic stresses.

## Figures and Tables

**Table 1:** Growth parameters of non-calamine (N-Cal) and calamine (Cal) ecotypes of *Silene vulgaris* primed with 0.1 mM acetylsalicylic acid (ASA), hydrogen peroxide (H_2_O_2_), salicylic acid (SA), and nitric oxide (NO) prior to Cd exposure.

Priming Agent	MC ^1^	Shoot Dry Weight (g)	Specific Morphological Remarks after 6 Weeks of Cd Exposure
N-Cal ecotype			
Control (H_2_O)	8.1 ± 0.8 Bc	0.369 ± 0.03 Bc	Typical shoot morphology
ASA	7.5 ± 1.1 Bc	0.395 ± 0.02 Bc	Typical shoot morphology
H_2_O_2_	8.8 ± 1.4 Bc	0.217 ± 0.06 Cd	Narrow leaf blades, pale green
SA	11.4 ± 0.6 Ab	0.472 ± 0.03 Ab	Enlarged, wide leaf blades
NO	9.2 ± 0.3 Bc	0.441 ± 0.03 Abc	Intense branching, enlarged explants
Cal ecotype			
Control (H_2_O)	11.9 ± 1.3 Zb	0.538 ± 0.01 Ya	Typical shoot morphology
ASA	15.6 ± 0.3 Ya	0.554 ± 0.01 Ya	Typical shoot morphology
H_2_O_2_	13.6 ± 1.1 Zb	0.446 ± 0.03 Zbc	Red pigmentation
SA	15.2 ± 0.8 Ya	0.512 ± 0.04 Ya	Elongated internodes
NO	13.1 ± 0.5 Zb	0.577 ± 0.02 Ya	Flowering

^1^ MC—micropropagation coefficient. Values are means ± SD, *n* = 4. Significant differences between treatments within each ecotype are marked with capital letters (A–C for N-Cal, Y–Z for N-Cal) according to one-way ANOVA and post hoc Tukey test, whereas significant differences between treatments according to two-way ANOVA and post hoc Tukey test are marked with lowercase letters.

**Table 2:** Profile of protein carbonyl derivatives: aldehydes and ketones in the shoots of non-calamine (N-Cal) and calamine (Cal) ecotypes of *Silene vulgaris* primed with 0.1 mM acetylsalicylic acid (ASA), hydrogen peroxide (H_2_O_2_), salicylic acid (SA), and nitric oxide (NO) prior to Cd exposure.

Ecotype and Priming Treatment	Carbonyl Derivatives (nmol·mg^−1^ Protein)	
	Aldehydes	Ketones
N-Cal		
Control (H_2_O)	15.8 ± 3.0 Cd	8.7 ± 1.8 Cd
ASA	19.4 ± 2.3 BCcd	10.5 ± 1.2 BCcd
H_2_O_2_	21.4 ± 4.1 ABCc	12.2 ± 3.5 ABCc
SA	28.0 ± 2.6 Aab	16.0 ± 1.9 Aab
NO	24.2 ± 2.1 ABbc	13.6 ± 1.2 ABbc
Cal		
Control (H_2_O)	17.0 ± 1.7 Zd	7.6 ± 0.9 Zd
ASA	33.8 ± 1.6 Xa	18.2 ± 0.7 Xa
H_2_O_2_	23.7 ± 1.6 Ybc	13.3 ± 1.1 Ybc
SA	27.1 ± 2.8 Xb	14.0 ± 1.4 Xb
NO	29.4 ± 1.2 Xa	16.0 ± 0.7 Xa

Values are means ± SD (*n* = 3). Significant differences between treatments within each ecotype are marked with capital letters (A–C for N-Cal, X–Z for Cal) according to one-way ANOVA and post hoc Tukey test, whereas significant differences between species and treatments according to two-way ANOVA and post hoc Tukey test are marked with lowercase letters.

## Data Availability

Data are contained within the article and Supplementary Materials.

## Supplementary Materials

The following supporting information can be downloaded at: https://www.mdpi.com/article/10.3390/ijms242216075/s1.
