# Supplementary material for: Response to Cadmium in Silene vulgaris Ecotypes Is Distinctly Affected by Priming-Induced Changes in Oxidation Status of Macromolecules
Source: Int J Mol Sci. 2023 Nov 8;24(22):16075. doi: 10.3390/ijms242216075 (PMC10671773; doi:10.3390/ijms242216075)
Supplement: Supplementary file 1 [file ijms-24-16075-s001.zip › ijms-2691678-supplementary.pdf]

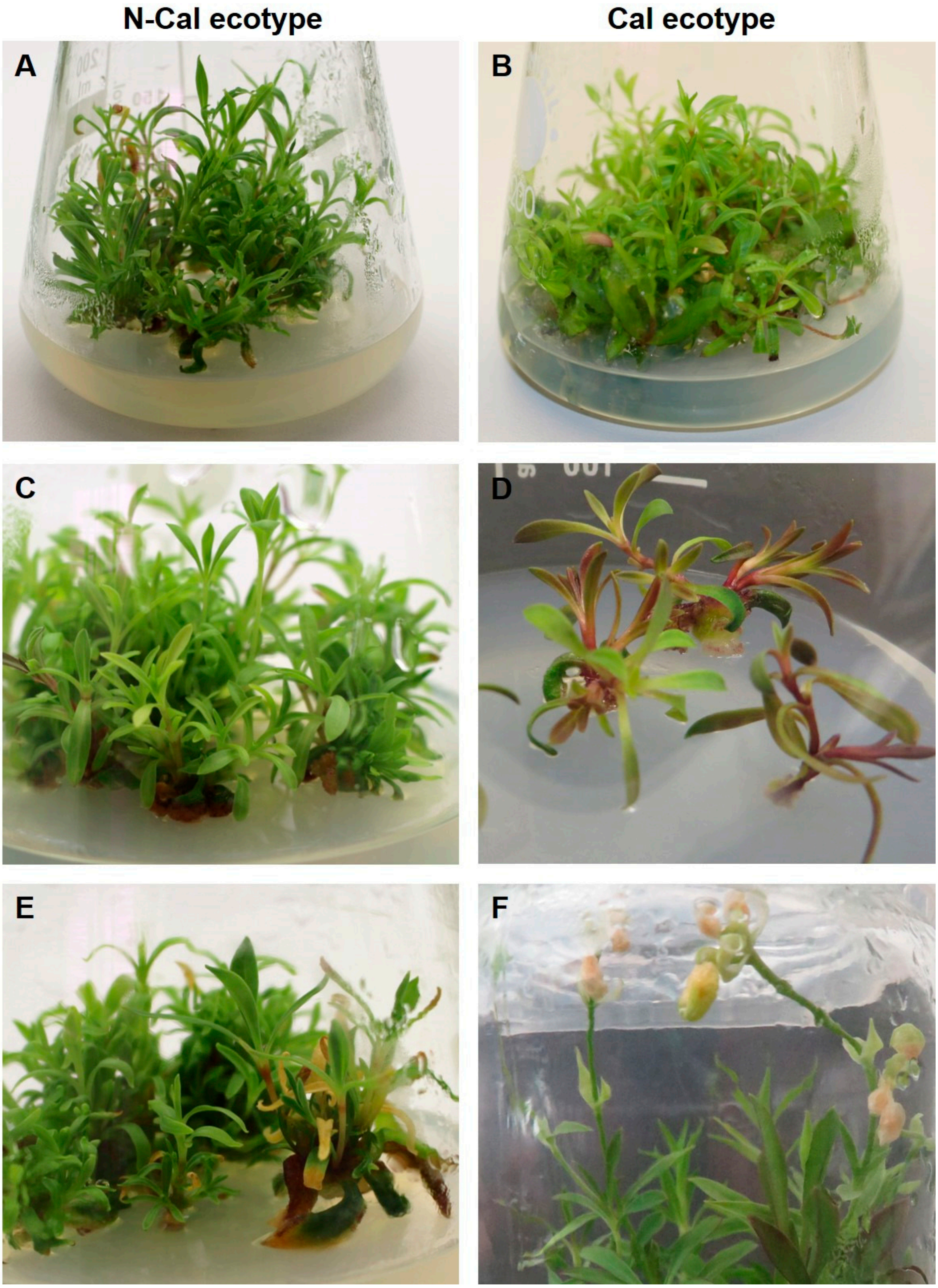

Figure S1. Specific morphological features of *S. vulgaris* ecotypes subjected to priming prior Cd exposure. (**A**,**B**)—control, non-primed plantlets; (**C**,**D**)—plantlets primed with H_2O_2, (**E**,**F**)—plantlets primed with NO.
